# Preoperative higher right ventricular stroke work index increases the risk of de novo aortic insufficiency after continuous-flow left ventricular assist device implantation

**DOI:** 10.1007/s10047-023-01411-1

**Published:** 2023-07-19

**Authors:** Shusaku Maeda, Koichi Toda, Kazuo Shimamura, Kei Nakamoto, Masataka Igeta, Yasushi Sakata, Yoshiki Sawa, Shigeru Miyagawa

**Affiliations:** 1 Department of Cardiovascular Surgery, Osaka University Graduate School of Medicine, 2-2 Yamadaoka, Suita, Osaka 565-0871 Japan; 2 Department of Cardiovascular Medicine, Osaka University Graduate School of Medicine, Suita, Osaka Japan; 3 Department of Biostatistics, Hyogo College of Medicine, Nishinomiya, Hyogo Japan

**Keywords:** Left ventricular assist device, Aortic insufficiency, Aortic valve opening, Right ventricular stroke work index, Shear stress

## Abstract

During continuous-flow left ventricular assist device (CF-LVAD) support, hemodynamic shear stress causes a burden on aortic valve (AV) leaflets, leading to de novo aortic insufficiency (AI). This study investigated the influence of preoperative hemodynamic parameters on de novo AI in CF-LVAD recipients. We reviewed 125 patients who underwent CF-LVAD implantation without concomitant AV surgery between 2005 and 2018. De novo AI was defined as moderate or severe AI in those with none or trivial preoperative AI. During mean 30 ± 16 months of CF-LVAD support, de novo AI-free rate was 86% and 67% at 1 and 2 years, respectively. Multivariable analysis showed that higher right ventricular stroke work index (RVSWI) (hazard ratio, 1.12 /g/m^2^/beat; 95% confidence interval, 1.00–1.20; *p* = 0.047) and trivial grade AI (hazard ratio, 2.8; 95% confidence interval, 1.2–6.4; *p* = 0.020) were independent preoperative risk factors for de novo AI. The longitudinal analysis using generalized mixed effects model showed that higher RVSWI was associated with continuous AV closure after LVAD implantation (Odd ratio, 1.20/g/m^2^/beat; 95% confidence interval, 1.00–1.43 /g/m^2^/beat; *p* = 0.047). Right heart catheterization revealed that preoperative RVSWI was positively correlated with postoperative pump flow index in patients with continuously closed AV (*r* = 0.44, *p* = 0.04, *n* = 22). Preoperative higher RVSWI was a significant risk factor for de novo AI following CF-LVAD implantation. In patients with preserved right ventricular function, postoperative higher pump flow may affect AI development via hemodynamic stress on the AV.

**Supplementary Information:**

The online version contains supplementary material available at 10.1007/s10047-023-01411-1.

## Introduction

De novo aortic insufficiency (AI) after continuous-flow left ventricular assist device (CF-LVAD) implantation is a common but significant complication, leading to heart failure and mortality [1–3]. For prevention of this serious complication, aortic valve (AV) closure or replacement at the time of device implantation may be needed in some patients [4–6]. On the other hand, concomitant AV surgery significantly prolongs operation time and increases operative risks [7]. Therefore, reliable preoperative risk factors for de novo AI are necessary when considering prophylactic AV surgery.

Previous studies have reported that older age, female sex, and small body surface area (BSA) are preoperative risk factors for AI development [1, 8]. Regarding surgical and postoperative factors, outflow graft anastomosis design [9–11], AV opening status [12–16], and CF-LVAD pump speed setting [14] were recognized as important factors associated with de novo AI. Its development in patients with a small BSA, continuously closed AV, unfavorable outflow graft anastomosis design, or higher pump speed setting may be explained by increased hemodynamic shear stress on the AV leaflets caused by CF-LVAD pump flow [1, 17–20]. Although hemodynamics stress may play an important role for de novo AI development, preoperative risk factor factors related to hemodynamics have not been investigated. The aim of this study was to investigate the influence of preoperative hemodynamic parameters on de novo AI development after CF-LVAD implantation.

## Patients and methods

### Study population

We reviewed 191 consecutive patients who underwent CF-LVAD implantation between 2005 and 2018. During that period, DuraHeart (Terumo Heart, Ann Arbor, MI, USA), EVAHEART (SunMedical, Suwa, Japan), HeartMate II (Thoratec, Pleasanton, CA, USA), Jarvik2000 (Jarvik Heart, NY, USA) and HeartWare (HeartWare, Framingham, MA, USA) devices were implanted at our institution. For all cases, device selection was made at the preoperative heart team conference. Twenty-three patients who underwent concomitant AV surgery and 15 who had preoperative mild AI without concomitant AV surgery were excluded, as were 28 with missing data regarding preoperative right heart catheter examination. Ethical approval was obtained from Osaka University Hospital Institutional Review Board (IRB number: 16105) and all patients and their family members provided informed consent to participate in related clinical studies before CF-LVAD implantation.

### Echocardiography

Two-dimensional and Doppler transthoracic echocardiographic examinations were performed at 1, 3, 6, and 12 months, and then every 6 months thereafter until heart transplantation, device explant, or death. De novo AI was defined as the first instance of moderate or severe AI in patients with no or trivial preoperative AI. AV opening status was evaluated visually and classified as continuously closed, or intermittently or fully opening with each beat.

### Right heart catheterization

Preoperative hemodynamic parameters were determined under stable conditions. Cardiac output was measured using Fick’s method, which is suitable for determining cardiac output in patients with severe heart failure or tricuspid regurgitation.21 Preoperative right ventricular (RV) function was quantified by RV stroke work index (RVSWI), right atrial pressure/pulmonary capillary wedge pressure (RAP/PCWP) ratio, and pulmonary artery pulsatility index (PAPi), which were calculated using the following equation: RVSWI = stroke volume index × (mean pulmonary artery pressure – mean RAP) × 0.0136; PAPi = (systolic pulmonary artery pressure–diastolic pulmonary artery pressure)/RAP; with stroke volume index calculated by cardiac index (CI) divided by heart rate. Postoperative right heart catheterization was performed in a limited number of the enrolled patients. Results of right heart catheterization performed > 200 days after CF-LVAD implantation or with the presence of de novo AI were excluded, because AI progression can reduce CI by affecting RV function [21].

### Computed tomographic image analysis

**Fig. 1:**
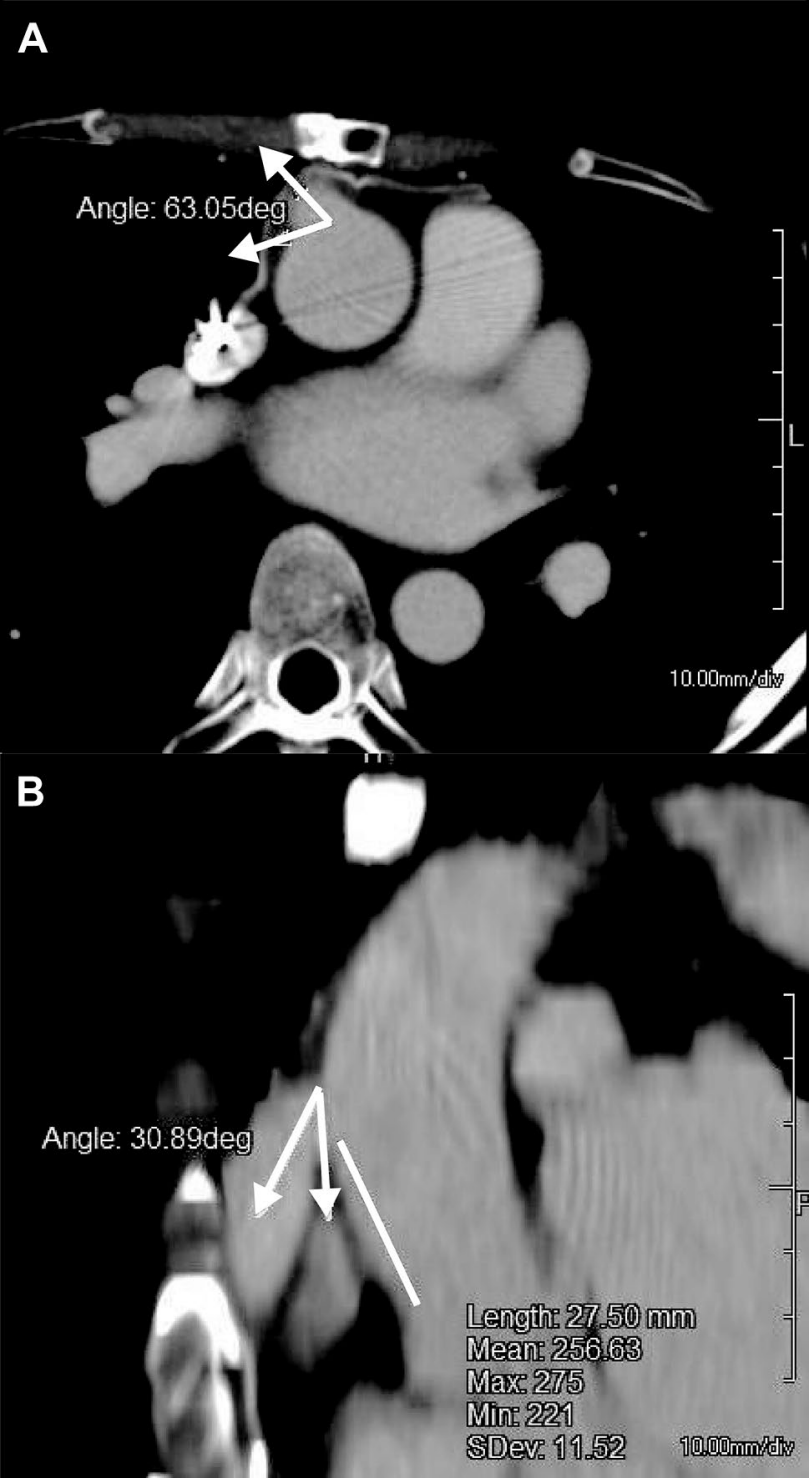
Representative computed tomographic images used to examine outflow graft anastomosis design. **A** Angle indicates azimuth angle. **B** Angle indicates inclination angle. Line indicates distance between sino-tubular junction and graft anastomosis location

The three-dimensional geometry of the outflow graft anastomosis design was extracted from computed tomography data. As previously described by Callington et al. [9], the azimuth angle was determined on the transversal plane (Fig. 1A). Similarly, the distance between the sino-tubular junction and graft anastomosis location, and inclination angle were measured on coronal planes (Fig. 1B).

### Device speed adjustment

At our institution, CF-LVAD pump speed was optimized prior to discharge based on symptoms, echocardiography findings, and/or right heart catheterization findings. In all of the present patients, attempts were made to gradually lower pump speed to promote AV opening, though that was not achieved in nearly half because of heart failure symptoms, insufficient left ventricular unloading, or higher PCWP.

### Statistical analysis

Continuous variables are summarized as mean values with standard deviation. Categorical variables are summarized as frequency with percentage. Survival rate and the rate of freedom from de novo AI were estimated using the Kaplan–Meier product limit method. Associations of preoperative and postoperative factors with de novo AI were examined using multivariable analysis with Cox proportional-hazards model. The relationships between preoperative RVSWI and categorical variables such as etiology, inotropic support, and intra-aortic balloon pumping were analyzed using one-way analysis of variance (ANOVA). The relationships between preoperative RVSWI and continuous variables such as left ventricular ejection fraction and pump flow index were analyzed using the univariable regression model and Pearson’s correlation coefficient. Association of preoperative RVSWI with postoperative AV opening closure was examined using a longitudinal mixed effect logistic regression model with preoperative RVSWI as a continuous variable, time (1, 3, 6, 12 months, 1, 2, and 3 years) as a categorial variable. Preoperative RVSWI and time were included as fixed effects and the intercept was included as a random effect. All *p*-values are two-sided and those < 0.05 were considered to indicate statistical significance. All statistical analyses were performed using the JMP Pro14 and SAS software package (SAS Institute, Cary, NC).

## Results

### Patient characteristics and clinical outcomes

A total of 125 CF-LVAD patients with none or trivial preoperative AI were enrolled, and their characteristics are summarized in Table 1. Age was 43 ± 14 years, 87 (70%) were male, and BSA was 1.61 ± 0.20 m^2^. During the study period, 16 patients died, 40 underwent heart transplantation after 33 ± 11 months of waiting, and 7 underwent CF-LVAD explantation. During the 30 ± 16 months of CF-LVAD support, survival rate was 93% at 1 year and 89% at 2 years (Supplemental Fig. 1).

**Table 1:** Patient characteristics

Variables	
Age, years	43 ± 14
Sex, male	87 (70%)
BSA, m^2^	1.61 ± 0.20
Diagnosis	
DCM	77 (62%)
dHCM	22 (18%)
ICM	12 (10%)
Other	14 (10%)
INTERMACS profile	
1	9 (7%)
2	47 (38%)
3	59 (47%)
4	8 (6%)
BTB	2 (2%)
Echocardiographic data	
LVEF, %	21 ± 9
LVDd, mm	69 ± 14
LVDs, mm	63 ± 14
AI grade	
None	86 (69%)
Trivial	39 (31%)
RHC data	
HR,/min	81 ± 16
mPA, mmHg	29 ± 10
PAWP, mmHg	20 ± 8
RAP, mmHg	7.9 ± 4.6
CI, L/min	2.0 ± 0.6
RVSWI, g/m^2^/beat	7.3 ± 3.8
RAP/PAWP	0.38 ± 0.20
PAPi	3.7 ± 2.8
Preop. Inotrope	114 (91%)
Preop. IABP	23 (18%)
Device	
Jarvik2000	21 (17%)
HeartMateII	51 (41%)
HVAD	13 (10%)
EVAHEART	17 (14%)
DuraHeart	23 (18%)

*AI* aortic insufficiency*, BSA* body surface area*, BTB* bridge-to-bridge*, CI* cardiac index*, DCM* dilated cardiomyopathy*, dHCM* dilated phase of hypertrophic cardiomyopathy*, HR* heart rate*, HVAD* HeartWare left ventricular assist device*, IABP* intra-aortic balloon pumping*, ICM* ischemic cardiomyopathy*, INTERMACS* interagency registry for mechanically assisted circulatory support*, LVDd* diastolic left ventricular dimension*, LVDs* systolic left ventricular dimension*, LVEF* left ventricular ejection fraction*, mPA* mean pulmonary artery pressure, *PAPi* pulmonary artery pulsatility index*, PCWP* pulmonary capillary wedge pressure*, RAP* right atrial pressure, *RHC* right heart catheterization*, RVSWI* right ventricular stroke work index

### Preoperative risk factors for de novo AI

Preoperative AI grade was none in 86 (69%) and trivial in 39 (31%) patients. During 20 ± 14 months of echocardiographic follow-up, 32 patients developed de novo moderate or severe AI after CF-LVAD implantation. The rate of freedom from de novo AI was 86% at 1 year and 67% at 2 years.

**Fig. 2:**
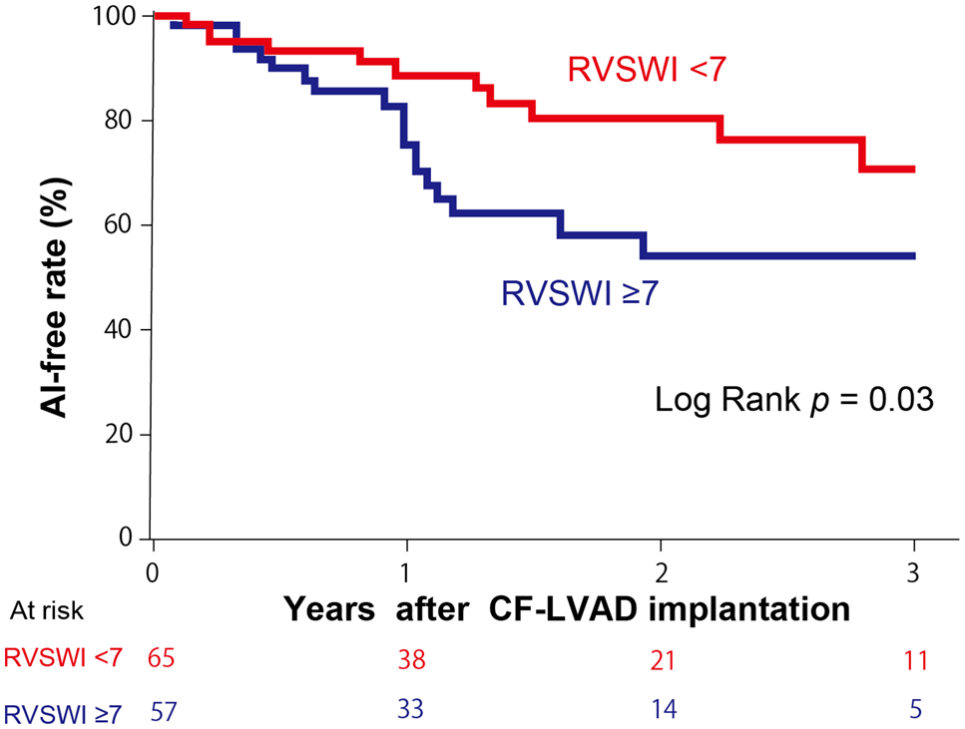
Kaplan–Meier estimates of freedom from de novo AI in lower preoperative RVSWI (< 7 g/m^2^/beat) patients versus higher RVSWI (≥ 7 g/m^2^/beat) patients

Multivariable analysis showed that higher preoperative RVSWI [hazard ratio (HR), 1.12 /g/m^2^/beat; 95% confidence interval (CI), 1.00–1.20 /g/m^2^/beat; *p* = 0.047] and preoperative trivial grade AI (HR, 2.8; 95% CI, 1.2–6.4; *p* = 0.020) were independent risk factors for de novo AI (Table 2). In the present cohort, the median RVSWI value was 6.8 g/m^2^/beat and rate for freedom from de novo AI was significantly higher in patients with preoperative RVSWI < 7 g/m^2^/beat (*p* = 0.03) (Fig. 2).

**Table 2:** Preoperative risk factors for de novo AI

Variable	Univariable	*p* value	Multivariable	*p* value
	HR (95% CI)		HR (95% CI)	
Age, per year	1.00 (0.97–1.02)	0.87		
Male	0.57 (0.28–1.16)	0.13	0.5 (0.2–1.3)	0.33
BSA, per m^2^	0.095 (0.021–0.49)	< 0.01	0.3 (0.1–2.0)	0.076
AI grade > none	1.7 (0.8–3.7)	0.16	2.5 (1.1–5.6)	0.020
RVSWI, per g/m^2^/beat	1.13 (1.03–1.21)	< 0.01	1.12 (1.02–1.22)	0.047
CVP/PCWP	0.39 (0.043–2.7)	0.36		
PAPi	1.1 (0.96–1.2)	0.20		
mPAP, per mmHg	0.98 (0.95–1.02)	0.39		
CVP, per mmHg	0.98 (0.90–1.05)	0.54		
Cardiac index, per L/min	1.70 (0.93–3.00)	0.09		
LVEF, per %	1.03 (0.99–1.07)	0.18		
Etiology		0.93		
Inotropic support	0.56 (0.17–1.83)	0.37		

*AI* aortic insufficiency, *BSA* body surface area, *CI* confidence interval, *HR* hazard ratio*, LVEF* left ventricular ejection fraction*, mPAP* mean pulmonary artery pressure, *PAPi* pulmonary artery pulsatility index*, PCWP* pulmonary capillary wedge pressure*, RAP* right atrial pressure*, RVSWI* right ventricular stroke work index

On the other hand, other risk factors for de novo AI, including age, female sex, and BSA, were not statistically significant [1, 8]. Other preoperative hemodynamic parameters, including RAP/PCWP, PAPi, mPAP, CVP, and CI were not also associated with de novo AI (Table 2). In our study cohort, etiology (*p* = 0.02) and preoperative intra-aortic balloon pumping (*p* = 0.02) was associated with preoperative RVSWI (Supplementary Table 1), but those factors were not risk factors of de novo AI development (Table2). Inotropic support (*p* = 0.32) was not associated with RVSWI and left ventricular ejection fraction (*r* = 0.21, *p* = 0.02) was not correlated with RVSWI.

### Outflow graft anastomosis design

Postoperative computed tomographic images were available for 112 (90%) patients. The mean distance between the sino-tubular junction and graft was 24 ± 5 mm, while inclination angle was 58 ± 17° and azimuth angle was 105 ± 20°. These geometrical parameters related to outflow graft anastomosis were not associated with de novo AI development (Table 3).

**Table 3:** Outflow graft anastomosis design and association with de novo AI

Variable	Value	Univariable	*p* value
		HR (95% CI)	
Distance between STJ and graft, mm	24 ± 5	0.97 (0.89–1.05)	0.48
Inclination angle, degrees	58 ± 17	1.01 (0.99–1.03)	0.21
Azimuth angle, degrees	105 ± 20	0.99 (0.98–1.01)	0.48

*AI* aortic insufficiency, *STJ* sino-tubular junction

### Pump speed settings

CF-LVAD pump speed settings at discharge are shown according to the device in Table 4. In all devices, pump speed settings seemed to be similar or lower as compared with previous reports probably because of small BSA of our patients [13, 16, 22, 23]. Associations between pump speed settings and de novo AI development were difficult to be statistically analyzed because of the low number of patients with each device. Among the devices, HeartMate II was implanted in a relatively large number of patients (*n* = 48), but hazard ratio per 100 rpm was approximately 1.0 (Supplementary Table 2).

### AV opening status

In the present cohort, the rate of continuous AV closure at 1 month was 78%, while those at 3, 6, 12, and 24 months were approximately 65%. The rate of AI development was compared between patients with a continuously closed AV in all echocardiographic follow-up (*n* = 65) and those showing AV opening at least once (*n* = 59). Cox proportional-hazards model showed that continuously closed AV was associated with de novo AI (HR, 2.2; 95% CI, 1.2–6.4; *p* = 0.043).

### Impact of preoperative RVSWI on AV opening status

Since higher RVSWI was the significant risk factor for de novo AI, we examined the influence of preoperative RVSWI on AV opening status under LVAD support. The longitudinal analysis using generalized mixed effects model showed that higher preoperative RVSWI was associated with continuous AV closure after LVAD implantation (Odd ratio, 1.20/g/m^2^/beat; 95% CI, 1.00–1.43/g/m^2^/beat; *p* = 0.047) (Supplementary Table 3).

### Relationship between preoperative RVSWI and postoperative pump flow rate

De novo AI development in patients with higher preoperative RVSWI (Table 2, Fig. 2) may be explained by greater pump flow rate, which could impose a higher hemodynamic shear stress on AV leaflets. However, pump flow rate cannot be accurately estimated by the pump power consumption [24]. Furthermore, even if determined with right heart catheterization, accurate measurement of pump flow rate is impossible in patients with AV opening or significant AI because of antegrade or retrograde flow through the AV. Therefore, the relationship between preoperative RVSWI and postoperative pump flow rate was examined in patients with a continuously closed AV and without significant AI, in whom pump flow rate was equivalent to cardiac output measured with right heart catheterization.

**Fig. 3:**
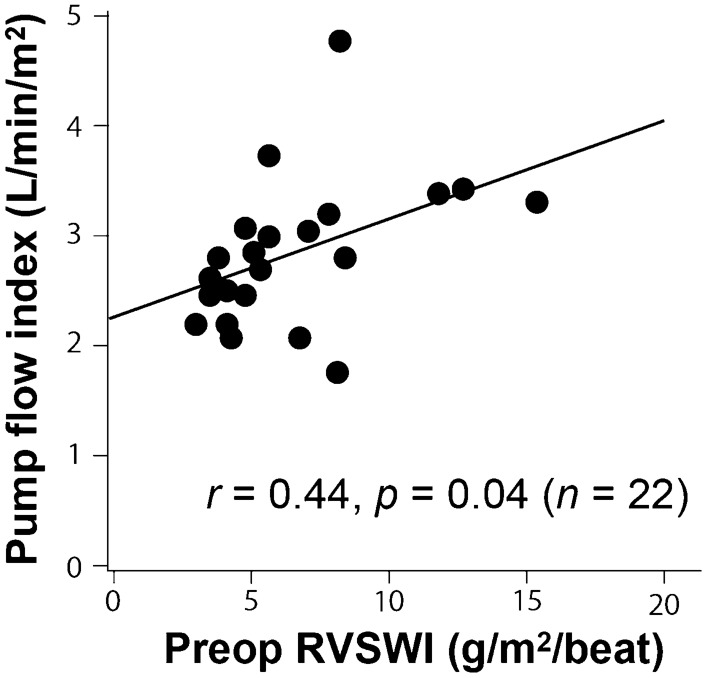
Relationship between preoperative RVSWI and CF-LVAD pump flow index in patients with continuously closed AV

Postoperative right heart catheterization was performed in 22 patients (18%) who had a continuously closed AV and no significant AI. At 88 ± 52 days after CF-LVAD implantation, pump flow index, which is equivalent to CI, was 2.8 ± 0.7 L/min/m^2^ in patients with preoperative RVSWI of 6.7 ± 3.2 g/m^2^/beat. Pearson’s correlation analysis revealed that pump flow index was positively correlated with preoperative RVSWI (*r* = 0.44, *p* = 0.04) (Fig. 3).

## Discussion

Although the mechanisms of AI development during CF-LVAD support remain to be fully elucidated, previous studies have suggested that non-physiologic hemodynamic stress caused by continuous pump flow plays an essential role [1]. In the present study, we focused on preoperative hemodynamics, which may influence hemodynamic stress on the AV. Our results demonstrated that higher preoperative RVSWI and preoperative trivial AI were significant risk factors for de novo AI after CF-LVAD implantation. Although we performed literature research, we were not able to find a study analyzing the relation between preoperative hemodynamics and de novo AI after CF-LVAD implantation, and our finding was unexpected because preserved preoperative RV function indicated by higher RVSWI has been recognized as a predictor of favorable outcome after CF-LVAD implantation, including lower incidence of RV failure, stroke, blood stream infection, and mortality [25–29]. However, higher CF-LVAD pump flow rate was expected in patients with higher preoperative RVSWI, thus we assumed that the association between de novo AI development and higher preoperative RVSWI could be explained by greater hemodynamic stress on the AV leaflets imposed by higher pump flow in patients with preserved RV function. Therefore, we investigated the relationship between pump flow rate and preoperative RVSWI, revealing a significant positive correlation between them. This result suggests that greater shear stress on the AV leaflets imposed by higher pump flow in patients with preserved RV function has a key role in development of de novo AI after CF-LVAD implantation.

The importance of shear stress on AV leaflets caused by retrograde CF-LVAD pump flow has been investigated by our group [18–20] as well as others [9–11]. We have found that retrograde pump flow towards the AV was higher in patients with de novo AI. However, retrograde pump flow towards the AV is influenced not only by total pump flow rate, but also location and angle of outflow graft anastomosis. Therefore, the impact of outflow graft design on the development of de novo AI was examined in this study. In the present cohort, neither location nor angle of outflow graft anastomosis was a predictor of de novo AI, possibly because of the favorable anastomosis design in most of these cases as compared with geometric data reported by Callington et al. [9].

AV opening status during CF-LVAD support has been recognized as an important predictor of de novo AI and was confirmed in the present study. Although continuous or intermittent AV opening was not achieved in more than half, attempts were made to gradually lower pump speed to promote valve opening in our institution. Therefore, pump speed was set at a relatively lower level in comparison with previous reports, which may explain no association between pump speed and de novo AI development in our cohort. These findings suggested that de novo AI in our patients was not caused by excessive LV unloading with a higher pump speed setting.

Our patients were relatively young (mean age, 43 years), and showed less ischemic cardiomyopathy (10%) and smaller BSA (mean BSA, 1.6 m^2^) as compared with those in other studies [8], thus their aortic root might be smaller than those in previous studies. In patients with a smaller aortic root, CF-LVAD pump flow may impose greater shear stress on the aortic root because aortic wall shear stress is inversely proportional to the dimension of the aorta [30]. This may explain why a small BSA was a significant risk factor for de novo AI as well as the higher occurrence of de novo AI in the present cohort as compared to patients with a larger BSA [2, 17]. In our cohort, preserved RV function with was a significant risk factor for de novo AI probably because of higher pump flow rate. However, this finding may not be applicable to larger size patients with a larger aortic root. Additional studies that focus on flow dynamics and shear stress in aortic roots with different sizes may be necessary to fully elucidate the mechanism of de novo AI after CF-LVAD implantation.

For prevention of de novo AI and its subsequent complications, prophylactic AV surgery at the time of device implantation may be needed in selected patients [1, 4–6]. The present findings showing a higher incidence of de novo AI in a higher RVSWI suggest that prophylactic AV surgery may be beneficial for patients with preserved RV function, who are expected to have a better postoperative outcome and may be more tolerable to concomitant AV surgery [10, 28, 29]. Furthermore, in the present study, preoperative trivial AI was the independent risk factor for de novo AI. Taken together, our results suggest that we may need to be more aggressive for prophylactic AV procedures in patients with preoperative AI that is equal or more than trivial, when long-term CF-LVAD support is required and preoperative RV function is preserved.

### Study limitations

The present study has some limitations, including its retrospective design performed for a greater than ten-year period. However, data for outflow graft anastomosis and pump speed setting were similar throughout the study period (data not shown). Furthermore, five different CF-LVAD devices were employed in the present cohort, thus the association between pump speed settings and de novo AI was examined in limited numbers of patients with the same device. Finally, postoperative right heart catheterization data were available for only a relatively small number of the examined cases. Further studies are warranted to confirm the relationships between preoperative RV function, pump flow rate, and de novo AI development.

## Conclusions

Preoperative higher RVSWI was a significant risk factor for de novo AI after CF-LVAD implantation. In patients with preserved RV function, postoperative higher pump flow may affect development of AI via hemodynamic stress on the AV.

### Supplementary Information

Below is the link to the electronic supplementary material.

Supplementary file1 (PDF 137 KB)

## Data Availability

Datasets for this study are available from the corresponding author upon reasonable request.
