# Supplementary material for: Preoperative higher right ventricular stroke work index increases the risk of de novo aortic insufficiency after continuous-flow left ventricular assist device implantation
Source: J Artif Organs. 2023 Jul 19;27(3):222–9. doi: 10.1007/s10047-023-01411-1 (PMC11345319; doi:10.1007/s10047-023-01411-1)
Supplement: Supplementary file 1 — Supplementary file1 (PDF 137 KB) [file 10047_2023_1411_MOESM1_ESM.pdf]

Supplemental Fig.1 Survival after CF-LVAD implantation.

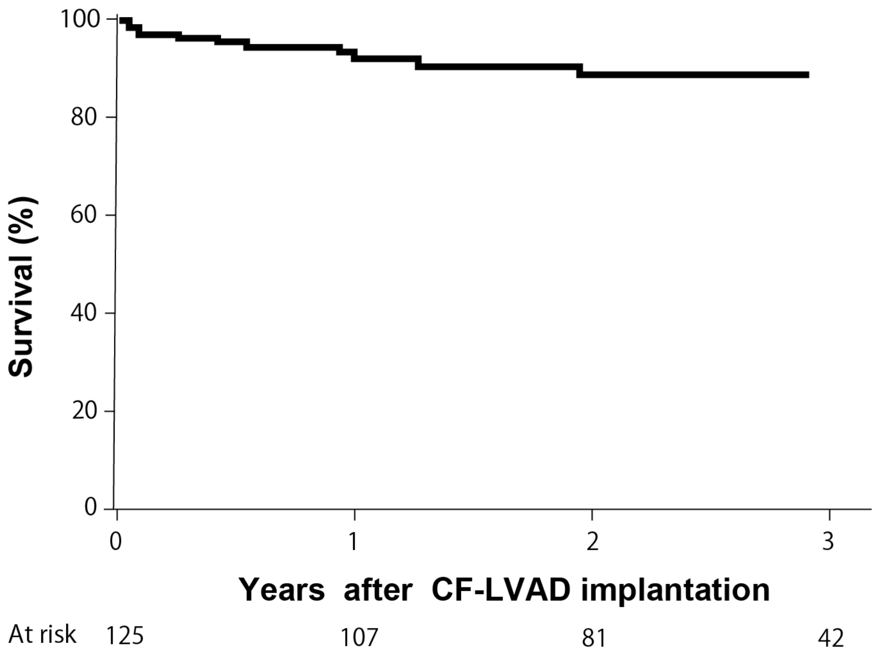

Supplementary Table 1 Preoperative RVSWI

	RVSWI, g/m^2/beat
Etiology	
DCM (*n* = 77)	7.1 ± 2.5
dHCM (*n* = 22)	7.6 ± 5.4
ICM (*n* = 12)	9.9 ± 5.8
other (*n* = 14)	5.5 ± 3.5
Preoperative IABP	
yes (*n* = 23)	6.0 ± 2.4
no (*n* = 102)	7.5 ± 4.0

DCM dilated cardiomyopathy, *dHCM* dilated phase of hypertrophic cardiomyopathy, *IABP* intra-aortic balloon pumping, *ICM* ischemic cardiomyopathy, *RVSWI* right ventricular stroke work index

Supplementary Table 2 CF-LVAD pump speed settings and association with de novo AI

		Univariable	
Device	Value	HR (95% CI)	*p*-Value
DuraHeart, rpm	1664 ± 89 (*n* = 23)	0.77 (0.39–1.52) / 100 rpm	0.58
EVAHEART, rpm	1760 ± 87 (*n* = 15)	0.67 (0.25–1.82) / 100 rpm	0.67
HeartMate II, rpm	8625 ± 282 (*n* = 48)	1.00 (0.97–1.02) / 100rpm	0.26
HVAD, rpm	2636 ± 150 (*n* = 9)	1.16 (0.58–2.32) /100 rpm	0.20
Jarvik 2000, rpm	9357 ± 497 (*n* = 14)	1.83 (0.50–6.71) / 1000 rpm	0.83

AI aortic insufficiency, *CF-LVAD* continuous-flow left ventricular assist device, *HVAD* HeartWare left ventricular assist device, *RVSWI* right ventricular stroke work index

Supplementary Table 3 Risk for continuous AV closure with the longitudinal analysis using generalized mix effect model

Variable	Estimate	Standard error	Odd ratio (95% CI)	*p*-Value
RVSWI, /g/m^2/beat	0.18	0.088	1.20 (1.00–1.43)	0.046
Time,				0.147
1 month/3 years	0.035	0.83	1.04 (0.20–5.36)	0.966
3 month/3 years	-0.57	0.65	0.57 (0.16–2.04)	0.384
6 month/3 years	-1.14	0.69	0.32 (0.08–1.24)	0.099
1 year/3 years	-1.15	0.64	0.32 (0.09–1.12)	0.075
2 years/3 years	-0.61	0.53	0.55 (0.19–1.53)	0.249

AV aortic valve, *RVSWI* right ventricular stroke work index
